# Depression Induced by CUMS Leads to Bladder Cancer Development and Local Tumor Immunosuppression in Mice

**DOI:** 10.1155/2021/5537523

**Published:** 2021-08-12

**Authors:** Zhiyu Qian, Weihong Ding, Qidong Zhou, Chuanyu Sun, Ke Xu

**Affiliations:** Department of Urology, Huashan Hospital Fudan University, Shanghai, China

## Abstract

Depression is a common mental disease in bladder cancer patients, leading to a loss of happiness, an increase in the suicide rate, and higher mortality. However, the influence of depression on bladder tumor tissue remains unknown. In this current study, a subcutaneous bladder cancer xenograft model was established on male C57 mice with mouse bladder cell line MB49. Chronic unpredictable mild stress (CUMS) was established to simulate depression in bladder cancer patients. The depression caused by CUMS was confirmed by testing sucrose preference and plasma cortisol and adrenocorticotropic hormone (ACTH) levels. Then, we measured and weighed tumors to demonstrate the promotion of tumor growth by CUMS. Immune-related cells and molecules were examined to reveal the mechanism. There is a significant decrease of CD8^+^/CD4^+^T cells ratio, NK cells, IL-2, and IFN-*γ* and a significant increase of T regs, IL-6, IL-1*β*, TNF-*α*, IL-10, and PGE2 in CUMS group, indicating the inhibition of immunity in the tumor microenvironment. Our results supported the perspective that depression exacerbated bladder cancer and revealed a possible mechanism. We suggest attaching importance to the psychological health of bladder cancer patients to prevent a worse prognosis induced by depression.

## 1. Introduction

Bladder cancer is the 10^th^ most common malignant tumor in the world. 550,000 newly diagnosed cases occurred and 200000 cases died every year [1, 2]. Bladder cancer not only causes physical damage but also brings about psychological distress. The psychological disorder in patients with bladder cancer is much more common than that of normal people [3, 4], which may be due to surgical suffering, decreased quality of life, or repeated invasive examination [5–7].

Depression is the most common mental disease in bladder cancer patients, which can lead to the loss of happiness and an increase in the suicide rate [8, 9]. The meta-analysis also demonstrated that the disease attributable mortality of bladder cancer patients with depression was 2.2 times higher, which is the highest among all kinds of cancer, than that of bladder cancer patients without mental disorders [10], which proved that depression can result in the deterioration of bladder cancer. A large number of clinical studies supported this view, but few researchers focused on how depression affected bladder tumor tissue.

Depression has complex and important effects on human immune function, such as a decreased function of natural killer cells (NK cells) [11], decreased ratio of CD8^+^/CD4^+^T cells [12, 13], and abnormal concentration of immune-related cytokines [14–16]. Bladder cancer carries one of the highest mutational loads among tumors [17] and is highly immunogenic. Therefore, bladder cancer relates closely to immune function and immunotherapy plays an important role in the treatment of bladder cancer [18], so it is important to study the influence of depression on the immune status in the tumor microenvironment.

Therefore, in the current study, we applied the CUMS model, a robust depression-inducing protocol for rodents [19], to C57 male mice with subcutaneous xenograft model of bladder cancer and examined the level of immune cells and cytokine to explore the mechanism of depression affecting the development of bladder cancer.

## 2. Methods

We partly followed the methods of our previous study [20].

### 2.1. Materials

Mouse ACTH ELISA Kit, mouse CORT ELISA Kit (Shanghai Lengton Bioscience Co., LTD), Mouse IL-2 ELISA Kit, Mouse IFN-*γ* ELISA Kit, Mouse IL-10 ELISA Kit, and prostaglandin E2/PGE2 Competitive ELISA Kit (MULTISCIENCES (LIANKE) BIOTECH, CO., LTD, China) were used. Anti-mouse CD4 antibody, anti-mouse CD8 antibody, and anti-mouse FOXP3 (BD Biosciences, Maryland, USA) antibody were used. All reagents and chemicals were of analytical grade.

### 2.2. Cell Lines and Animals

MB49 cells were cultured in RPMI 1640 medium supplemented with 10% fetal bovine serum (FBS) and 1% penicillin–streptomycin (10,000 U/mL) (Gibco, Life Technologies, Grand Island, New York, USA) in 5% CO_2_ at 37°C.

Male C57 mice weighing 9.3–11.4 g (3 weeks old, *n* = 20) were purchased from the Shanghai SLAC Laboratory Animal Co., Ltd. (Shanghai, China) and were raised under specific pathogen-free conditions. All animal experiments were approved by the animal ethics committee of Fudan University (Ethical approval number: 2019-HSYY-JS-240) and conformed to the guidelines of the National Research Council for Laboratory Animal Care in Research.

### 2.3. Subcutaneous Bladder Cancer Xenograft Model

Mice were group-housed under specific pathogen-free conditions and were allowed to adapt to the laboratory environment 1 week before the start of the experiments. MB49 cells harvested after brief incubation in 0.25% trypsin and 0.02% EDTA were injected subcutaneously into the right lateral chest wall of C57 mice at a concentration of 5 × 10^6^ cells/0.2 mL PBS each mouse. The puncture site was disinfected with alcohol before and after injection.

### 2.4. Chronic Unpredictable Mild Stress Model

Mice were randomly divided into CUMS (*n* = 10) or control (*n* = 10) groups after the in vivo implantation of tumor cells. The CUMS procedure was conducted according to previous studies [21]. Stress regimes in the current study consisted of the following eight stressors: food deprivation (24 h), water deprivation (24 h), tail pinch (5 min, 1 cm from the distal portion of the tail), forced swimming (3 min, at room temperature), physical restraint (2 h), soiled bedding (6 h), cage tilting (12 h), and crowded housing (12 h). Two random stressors were conducted daily for consecutive 15 days, with the same stressor not applied on two consecutive days. Mice in the control group were not subjected to any of these stressors. The sizes of tumors were measured with calipers every 3 days in each mouse. All of the mice were weighed and sacrificed by decapitation 15 days after tumor implantation. The removed tumors were weighed, and the tumor and blood samples were collected for further analysis.

### 2.5. Measurement of Cortisol and ACTH

The blood samples of mice were all collected before sacrifice at approximately 9:00 a.m. when mice were under abdominal anesthesia by 10% hydro-chloro-aldehyde. Plasma was separated by centrifugation and stored at −80°C. Samples were tested using enzyme-linked immunosorbent assay (ELISA) kits according to the instructions. The absorbance was measured at 450 nm by using a microplate reader (Labsystems Multiskan MS, 352).

### 2.6. Measurement of Cytokines

Mice of CUMS and control groups were, respectively, divided into two subgroups. The tumors of mice in the first subgroups (*n* = 5 in the CUMS group and *n* = 5 in the control group) were used to determine cytokines in the tumor. Tumor tissues were prepared into homogenate for ELISA after being washed, weighed, cut and fully homogenized. Interleukin-2 (IL-2), interferon gamma (IFN-*γ*), interleukin-10 (IL-10), and prostaglandin E2 (PGE2) were measured using ELISA kits. The absorbance was measured at 450 nm by using a microplate reader.

### 2.7. Histology and Immunohistochemical Assay

The tumors of mice in the second subgroups (*n* = 5 in the CUMS group and *n* = 5 in the control group) were used for histology and immunohistochemical assay. Bladder tumor xenografts were removed, fixed in 10% formalin, and embedded in paraffin. Sections were stained with hematoxylin and eosin (HE) to evaluate the histological changes using a light microscope (H550S, Nikon, Japan). In addition, immunohistochemical analysis of these sections was performed by using specific antibodies for CD4, CD8, CD56, FOXP3, IL-6, IL-1*β*, and TNF-*α*. After the color reaction, four random fields at 40x magnification were examined for each section to quantify target positive cells and total cells, and the results were presented as ratios of positive cells/total cells.

### 2.8. Statistical Analysis

STATA software (version 13.0) was used to analyze the experimental data. The data were presented as the mean ± SD and analyzed by a two-tailed *t*-test between two groups. A *p* value less than 0.05 was considered statistically significant.

## 3. Results

### 3.1. Introduction of CUMS in C57 Mice with Subcutaneous Bladder Cancer Xenografts

A sucrose preference test was performed on day 0 and day 15 separately to determine whether the CUMS model promoted depression in mice. The test is a widely used method to evaluate depressive symptoms, and the quantification is based on the formula: sucrose preference = [1% sucrose intake/total fluid intake] × 100%. There was no significant difference between the two groups on day 0, while the sucrose preference rate of CUMS group was significantly lower than that in the control group on day 15, demonstrating the depressive behavior of mice in CUMS group (Table 1).

**Figure 1:**
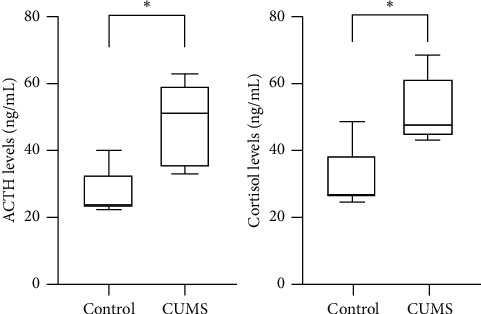
Cortisol and ACTH levels in both groups. CUMS increased plasma cortisol and ACTH in mice. ^*∗*^*p* < 0.05, CUMS group vs control group.

Meanwhile, blood samples were obtained after intraperitoneal anesthesia before sacrifice and plasma cortisol and ACTH levels were detected by ELISA. The concentration of plasma cortisol was 31.48 ± 9.92 ng/mL in the control group and 52.48 ± 10.20 ng/mL in the CUMS group, and the concentration of plasma ACTH was 26.99 ± 7.30 ng/mL in the control group and 48.08 ± 12.61 ng/mL in the CUMS group. The plasma cortisol level and plasma ACTH level of CUMS group were both significantly higher than those of the control group (Figure 1), indicating an abnormal activation of the HPA axis, which also verified the effectiveness of CUMS model in inducing depression.

### 3.2. CUMS Promoted Subcutaneous Bladder Cancer Xenograft Growth in C57 Mice

**Figure 2:**
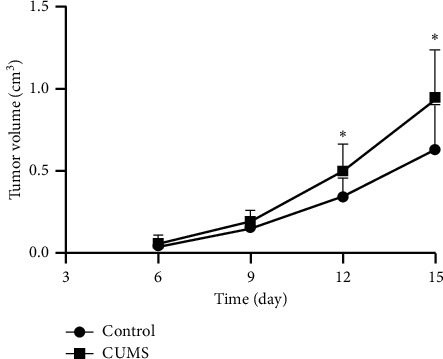
Tumor volumes measured at a 3-day interval. CUMS increases the tumor volumes of mice. ^*∗*^, *p* < 0.05 on day 12 and day 15, CUMS group vs control group.

Each mouse accepted a subcutaneous injection of MB49 cells at a concentration of 5 × 10^6^ cells/0.2 mL PBS on day 0. The tumors grew big and were visually distinct on day 5 or day 6 and grew rapidly since then. The sizes of tumors were measured every 3 days according to the formula [tumor volume = 0.5 ∗ length ∗ width ∗ width (day6, 9, 12); = 0.5 ∗ length ∗ width ∗ height (day15)]. On day 6 and day 9, there was no significant difference in the size of tumors between the two groups. However, the tumors of CUMS group were significantly larger than those of the control groups on day 12 and day 15, indicating that CUMS induced a more rapid tumor growth (Figure 2), which was also proved by weighing the bladder cancer xenografts after the experiment (Table 2). The tumor growth rate was 46.03% according to the formula [tumor growth rate = (Weight of tumors in the CUMS group − Weight of tumors in the control group) / Weight of tumors in the control group ∗ 100%]. Collectively, the depression of mice caused by CUMS promoted subcutaneous bladder cancer xenograft growth.

### 3.3. CUMS Model Modified the Quantity of Immune Cells

**Figure 3:**
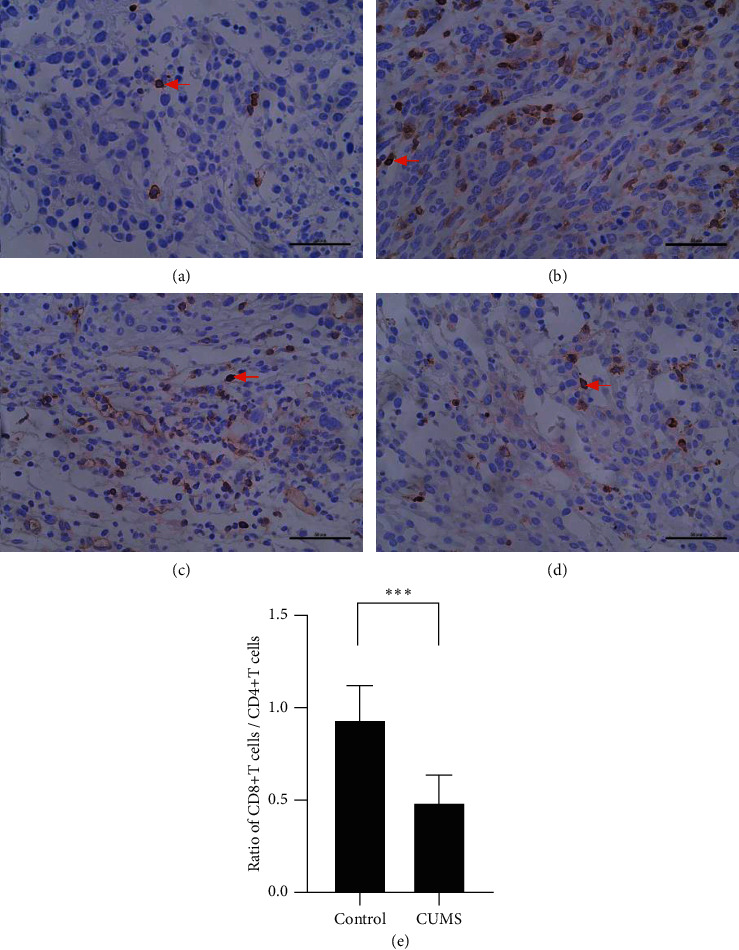
Immunohistochemical staining of CD4^+^ and CD8^+^T cells and the ratio of CD8^+^T cells/CD4^+^T cells. CUMS model reduced the ratio of CD8^+^T cells/CD4^+^T cells in TME. (a) Immunohistochemical staining targeting CD4 at 40x, control group. (b) Immunohistochemical staining targeting CD4 at 40x, CUMS group. (c) Immunohistochemical staining targeting CD8 at 40x, control group. (d) Immunohistochemical staining targeting CD8 at 40x, CUMS group. (e) The ratio of CD8^+^T cells/CD4^+^T cells in two groups. Red arrows indicated positive cells. ^*∗∗∗*^*p* < 0.01, the ratio of CD8^+^T cells/CD4^+^T cells, CUMS group vs control group.

CD8^+^T cells are the dominant anticancer cells in TME (tumor microenvironment) [22]. The number of CD8^+^T cells reflects local immune function and indicates the prognosis of cancer patients [23]. The ratio of CD8^+^T cells/CD4^+^T cells in the tumor microenvironment can reflect the specific immune responses to tumors. Immunohistochemistry was performed to analyze the proportion of CD8^+^T cells and CD4^+^T cells in the tumor. While the frequency of CD4^+^T cells in the two groups did not differ significantly, CD8^+^T cells in the CUMS group are significantly less than those in the control group (Table 3 and Figure 3), and a lower ratio of CD8^+^T cells/CD4^+^T cells was observed in the CUMS group, indicating that CUMS model can decrease the damage of cytotoxic T lymphocytes to bladder cancer cells.

**Figure 4:**
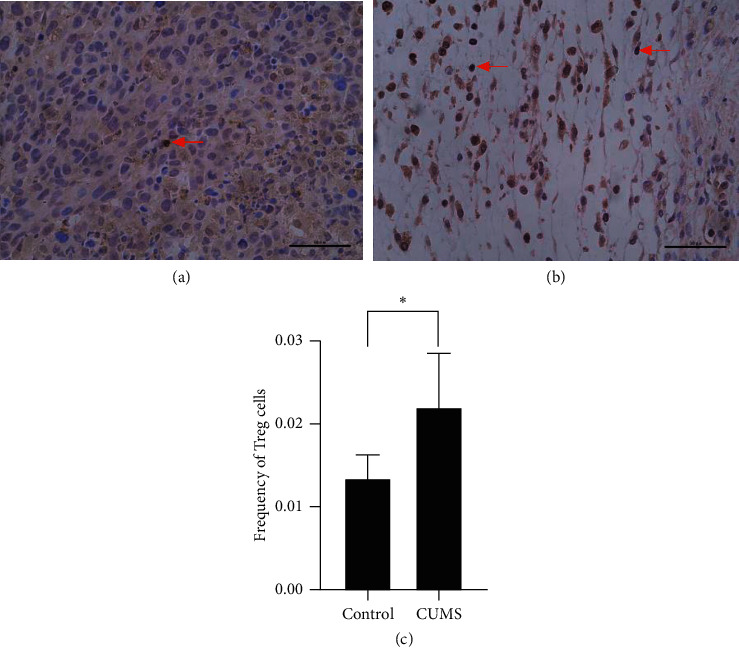
Immunohistochemical staining of T regs and the quantity of both groups. CUMS promoted the infiltration of T regs into tumor tissues. (a) Immunohistochemical staining targeting FOXP3 at 40x, control group. (b) Immunohistochemical staining targeting FOXP3 at 40x, CUMS group. (c) Frequency of T regs in tumor tissue of two groups. Red arrows indicated T regs. ^*∗*^, *p* < 0.05, frequency of T regs, CUMS group vs control group.

Regulatory cells (T regs) are a kind of T cells that play an important role in the development of tumors and highly express FOXP3. They infiltrate extensively around tumor tissues and develop an immunosuppressive TME [24], thus suppressing immune responses to tumors and ultimately leading to a worse prognosis of cancer patients [23]. Therefore, we also examined T regs. We found that the proportion of T regs in tumor tissues in the CUMS group was significantly higher than that in the control group, which indicated that CUMS promoted the infiltration of T regs into tumor tissue and decreased immune function (Figure 4).

**Figure 5:**
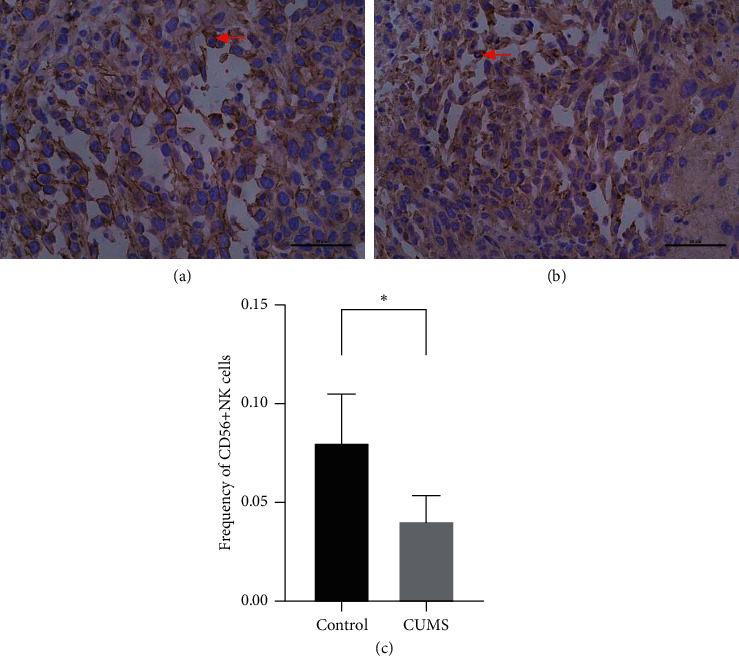
Immunohistochemical staining of NK cells and the quantity of both groups. CUMS decreased CD56^+^NK cells inside tumor tissues. (a) Immunohistochemical staining targeting CD56 at 40x, control group. (b) Immunohistochemical staining targeting CD56 at 40x, CUMS group. (c) Frequency of CD56^+^NK cells in tumor tissues of two groups. Red arrows indicated CD56^+^NK cells. ^*∗*^*p* < 0.05, frequency of CD56^+^NK cells, CUMS group vs control group.

NK cells can also mediate cytotoxicity, which is vital for tumor immunity. An increasing number of NK cells are associated with improved survival outcomes of bladder cancer patients [25]. CD56 is a surface antigen that occurs mostly in NK cells, so the frequency of NK cells was detected with CD56 antibody, and we found a significant difference between the two groups (Figure 5).

### 3.4. CUMS Model Modified the Quantity of Cells with High Expression of IL-6, IL-1*β*, and TNF-*α*

**Figure 6:**
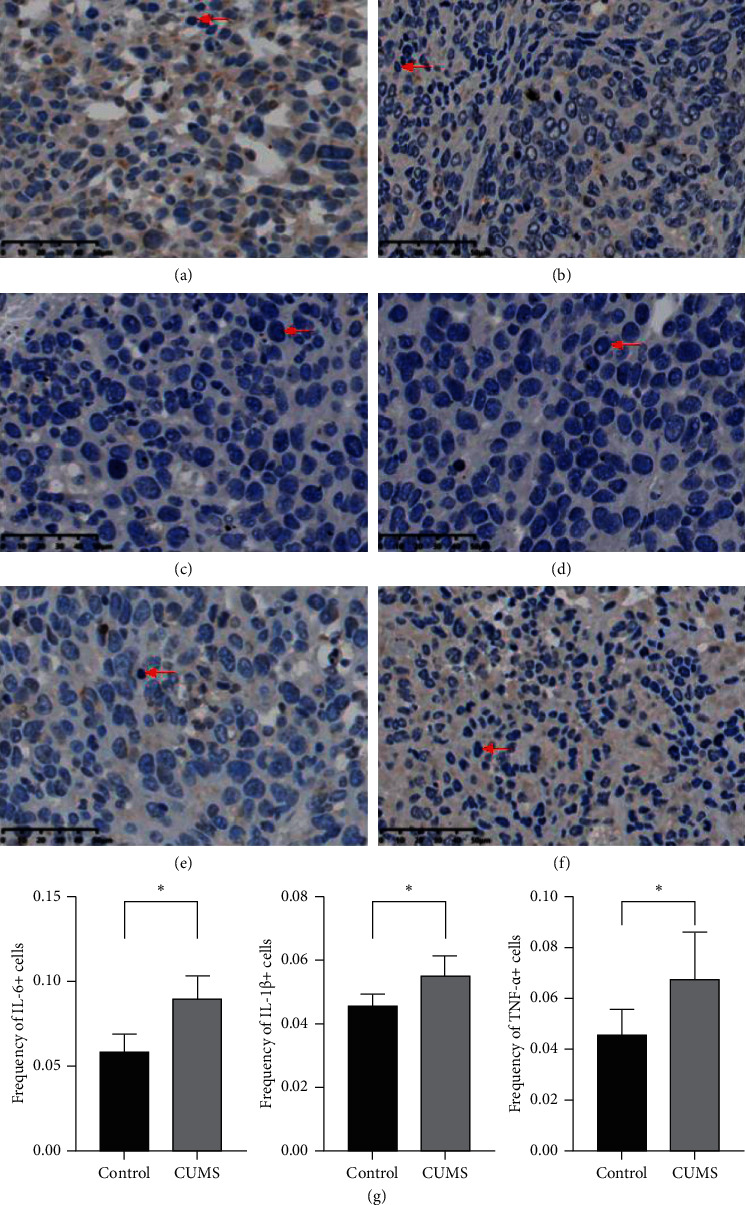
Immunohistochemical staining of IL-6, IL-1*β*, and TNF-*α*. CUMS model increased the expression of IL-6, IL-1*β*, and TNF-*α*. (a) Immunohistochemical staining targeting IL-6 at 40x, control group. (b) Immunohistochemical staining targeting IL-6 at 40x, CUMS group. (c) Immunohistochemical staining targeting IL-1*β* at 40x, control group. (d) Immunohistochemical staining targeting IL-1*β* at 40x, CUMS group. (e) Immunohistochemical staining targeting TNF-*α* at 40x, control group. (f) Immunohistochemical staining targeting TNF-*α* at 40x, CUMS group. (g) Frequency of positive cells of two groups. Red arrows indicated cells with high expression of IL-6, IL-1*β*, or TNF-*α*. ^*∗*^, *p* < 0.05, the ratio of positive cells and total cells, CUMS group vs control group.

IL-6, IL-1*β*, and TNF-*α* are 3 cytokines relevant to immunosuppression and tumor progression. We found that the ratio of cells with high expression of IL-6, IL-1*β*, or TNF-*α* is significantly higher in the CUMS group (Figure 6).

### 3.5. CUMS Model Modified the Level of Cytokines in Tumors

In addition to immune cells, immune-related cytokines in tumors were examined to explore how CUMS affected immune function.

**Figure 7:**
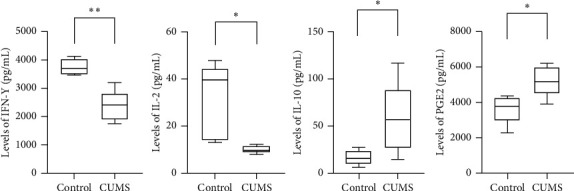
Levels of cytokines (IFN-*γ*, IL-2, IL-10, and PGE2) in tumors. CUMS model modified the level of molecules (IFN-*γ*, IL-2, IL-10, and PGE2) in tumors. ^*∗*^*p* < 0.05, CUMS group vs control group. ^*∗∗*^*p* < 0.01, CUMS group vs control group.

IFN-*γ* and IL-2 are the key markers of T-cell activation and proliferation, and it was found that the levels of them in CUMS group were both significantly lower than those in the control group. It suggested that CUMS could cause damage to T-cell function (Table 4 and Figure 7).

IL-10 and PGE2 are important molecules that inhibit immune function. We found that the levels of IL-10 and PGE2 in tumor tissues in the CUMS group were significantly higher compared with those in the control group, suggesting that CUMS could reduce the immune function to a lower level (Table 5 and Figure 7).

## 4. Discussion

Bladder cancer is a severe health problem worldwide. Among the newly diagnosed urothelial carcinoma patients, non-muscle-invasive bladder cancer (NMIBC) accounts for 70% [26], which is routinely treated by transurethral resection of bladder tumor (TURBT) with postoperative intravesical chemotherapy. Despite the fact that TURBT is a relatively less trauma operation, the NMIBC patients have poor mental health because of a series of invasive examinations and treatments after surgery, including re-TURBT, intravesical chemotherapy, regular cystoscopy, and urinary tract imaging [27]. MIBC (muscle-invasive bladder cancer) is advanced bladder cancer and its radical surgical treatment is radical cystectomy, which leads to a serious injury to patients and a significant decline in their quality of life as well as their psychological well-being [28].

It has been proved that depression can affect the immune status in peripheral blood, but it remains unknown whether the immune function in the tumor microenvironment can be modified by psychological disorders. Since bladder cancer is associated closely with immune function, the deterioration of the prognosis of bladder cancer patients with depression may be due to the altered immune status in the tumor microenvironment.

In this current study, the CUMS model has been applied to mice with subcutaneous bladder cancer xenograft in order to simulate depression in bladder cancer patients and examine the role of depression in bladder cancer. First, we confirmed the depression caused by CUMS through sucrose preference test and plasma cortisol and ACTH level, and then, we showed the promotion of tumor growth in the CUMS group. Moreover, we explored the possible mechanism by which depression worsened the prognosis of bladder cancer patients.

CD8^+^T cells are the major anticancer cells in the tumor microenvironment [29]. They mediated the cytotoxicity with FAS ligands and granzyme B. It has been proved that the presence of intratumoral cytotoxic CD8^+^T cells is associated with an improved prognosis in both non-muscle-invasive [30] and muscle-invasive bladder cancer [31]. CD4^+^T cells have bilateral effects on anticancer immunity, depending on the subsets. The ratio of CD8^+^T cells/CD4^+^T cells can influence the product regarding tumor eradication [32]. In bladder cancer, a larger ratio of CD8^+^T cell/CD4^+^T cell indicates a more rapid tumor growth and a better response to immunotherapy [33]. Our results showed a significant decrease in the ratio in CUMS group, indicating that depression can exert a negative influence on bladder cancer patients.

T regs play central roles in the maintenance of self-tolerance in healthy individuals [34]. However, in cancer patients, T regs constitute the immunosuppressive network to inhibit effective antitumor immunity, thereby promoting cancer progression [35]. T regs can be divided into naive T regs, effector T regs, and non-T regs. Effector T regs are the only fraction that possesses strong immunosuppressive activity, as well as the only fraction that highly expresses FOXP3. Thus, we performed immunohistochemistry to examine this subset of T regs. We found that the effector T regs are higher in the CUMS group, indicating a suppressed antitumor immunity.

NK cells, just like CD8^+^T cells, are important immune cells in TME with cytotoxic function. However, CD8^+^T cells require the presentation of antigens in the context of major histocompatibility complex class I molecules (MHC I) on the surface of tumor cells harboring mutated antigens to exert their cytotoxic function. When MHC I is downregulated by tumor cells, which is a common and important mechanism to evade immune surveillance, NK cells play a dominant role in antitumor defense [36]. Based on the presence of surface antigen CD56, NK cells can be divided into CD56^+^ or CD56^−^NK cells, and CD56^+^NK cells play a major role in cytotoxic function. Recent studies showed that NK cells could increase or decrease their own CD56 expression according to their immune status, thus regulating their own cytotoxic activity [37]. Therefore, we used CD56 antibody to quantify CD56^+^NK cells to evaluate the cytotoxic function of NK cells in the tumor microenvironment. Our results showed that CD56^+^NK cells decreased significantly in the CUMS group, indicating that the cytotoxic effect of NK cells was weakened.

IL-6 is an important molecule in IL-6/JAK/STAT3 signaling pathway. The activation of the IL-6/JAK/STAT3 signaling pathway can inhibit the function of immune cells in the tumor microenvironment, and inhibition of the IL-6/JAK/STAT3 signaling pathway can inhibit the growth of tumor cells and alleviate the immunosuppression in the tumor microenvironment [38]. Our results showed that CD6^+^ cells increased significantly in the CUMS group, indicating that the function of immune cells in CUMS group is suppressed.

Tumor necrosis factor-*α* (TNF-*α*) is a proinflammatory cytokine that can regulate cytokine production, cell survival, and cell death. Although TNF-*α* was used as a treatment for cancer at first, it is now considered to promote cancer by developing blood vessels, activating oncogene, and causing DNA damage [39]. In our study, we observed a significant increase in the expression of TNF-*α* in the CUMS group, suggesting that depression may promote tumor progression through TNF-*α*.

IL-1*β* is a common cytokine, which has a two-way effect on the tumor. However, its main function is to promote tumor progression, by promoting angiogenesis and tumor metastasis and suppressing immune function [40]. Our results showed that IL-1*β*^+^ cells increased significantly in the CUMS group, indicating that CUMS model has adverse effects on tumors.

IFN-*γ* is secreted predominantly by activated lymphocytes and regulated by extremely complex mechanisms. It can upregulate the MHC molecules [41, 42] as well as the upregulation of the whole MHC I and II antigen processing and presentation [43], which is crucial for cytotoxic T-cell activation. IFN-*γ* can also activate macrophages and NK cells to improve innate immunity [44]. IL-2 is a necessary cytokine for the proliferation and activation of T cells [45, 46], the promotion of cytotoxicity of NK cells [47], and the regulation of T regs function [48]. It has been developed as a medicine for melanoma, kidney cancer, and other cancer by enhancing immunity. IL-10 is a kind of cytokine mainly produced by immune cells. Its main function is to maintain the tolerance of T cells to autoantigens or harmless antigens, so it is the key negative regulatory cytokine of immunity. In malignant tumors, IL-10 can be secreted by T regs into the tumor microenvironment and adjacent lymph nodes, subsequently weakening local anticancer immunity [49]. PGE2 is a bioactive lipid that can elicit a wide range of biological effects. In the tumor microenvironment, it suppresses the cytotoxicity and cytokine production of NK cells [50] and promotes the proliferation and function of T regs [51]. In our experiment, IFN-*γ* and IL-2 were significantly decreased in tumors of the CUMS group, while the levels of IL-10 and PGE2 were significantly higher, indicating that CUMS caused an inhibition of antitumor immunity.

This current study focused on the influence of depression on the immune status of bladder cancer, including immune cells and molecules, and revealed the possible mechanism that depression promoted tumor growth. It is essential for clinicians to pay more attention to the mental health of bladder cancer patients, so as to avoid poor prognosis induced by psychological distress.

## Figures and Tables

**Table 1:** Sucrose preference in both groups on day 0 and day 15.

	Sucrose preference, day 0 (%, mean ± SD)	Sucrose preference, day 15 (%, mean ± SD)
Control group (*n* = 10)	73.2 ± 8.0	72.9 ± 8.0^*∗∗*^
CUMS group (*n* = 10)	72.6 ± 6.2	63.1 ± 8.4^*∗*^

A decreased sucrose preference was found in mice from the CUMS group. ^*∗*^*p* > 0.05, CUMS group vs control group on day 0; *p* < 0.05, CUMS group vs control group on day 15; ^*∗∗*^*p* > 0.05, control group on day 0 vs control group on day 15; *p* < 0.05, CUMS group on day 0 vs CUMS group on day 15.

**Table 2:** Weight of mice before and after experiments, weight of removed tumors, and tumor growth rate.

	Weight of mice (day 0)	Weight of mice (day 15)	Weight of tumors	Tumor growth rate (%)
Control group (g, mean ± SD, *n* = 10)	10.32 ± 0.63	19.31 ± 0.34	1.26 ± 0.37	
CUMS group (g, mean ± SD, *n* = 10)	9.79 ± 0.38^*∗*^	20.16 ± 1.43^*∗*^	1.84 ± 0.46^*∗∗*^	46.03

CUMS promoted the bladder tumor growth significantly. ^*∗*^There was no significant difference between the weights of mice in the two groups on day 0 or day 15. ^*∗∗*^Weights of tumors from the CUMS group were significantly higher than those from the control group.

**Table 3:** Frequency of CD4^+^T cells and CD8^+^T cells and the ratio of CD8^+^T cells/CD4^+^T cells.

	CD4^+^T cells (%)	CD8^+^T cells (%)	Ratio of CD8^+^T cells/CD4^+^T cells
Control group (*n* = 5, mean ± SD)	2.83 ± 0.38	2.53 ± 0.18	0.936 ± 0.082
CUMS group (*n* = 5, mean ± SD)	4.15 ± 0.50^*∗*^	1.91 ± 0.11^*∗∗*^	0.489 ± 0.066^*∗∗∗*^

Frequency of CD4^+^T cells and CD8^+^T cells was measured through immunohistochemistry, and the ratio was calculated. CUMS decreased the frequency of CD8^+^T cells and the ratio of CD8^+^T cells / CD4^+^T cells. ^*∗*^*p* > 0.05, frequency of CD4^+^T cells, CUMS group vs control group; ^*∗∗*^*p* < 0.05, frequency of CD8^+^T cells, CUMS group vs control group; ^*∗∗∗*^*p* < 0.01, the ratio of CD8^+^T cells/CD4^+^T cells, CUMS group vs control group.

**Table 4:** Levels of cytokines (IFN-*γ*, IL-2) in tumors.

	IFN-*γ* (pg/mL)	IL-2 (pg/mL)
Control group (*n* = 5, mean ± SD)	3752 ± 126	31.07 ± 7.23
CUMS group (*n* = 5, mean ± SD)	2372 ± 242^*∗∗*^	9.90 ± 0.68^*∗*^

CUMS model decreased the level of cytokines (IFN-*γ*, IL-2) in tumors. ^*∗*^*p* < 0.05, level of IL-2, CUMS group vs control group. ^*∗∗*^*p* < 0.01, level of IFN-*γ*, CUMS group vs control group.

**Table 5:** Levels of cytokines (IL-10, PGE2) in tumors.

	IL-10 (pg/mL)	PGE2 (pg/mL)
Control group (*n* = 5, mean ± SD)	17.34 ± 3.41	3648 ± 356
CUMS group (*n* = 5, mean ± SD)	57.15 ± 16.76^*∗*^	5222 ± 384^*∗*^

CUMS model increased the level of cytokines (IL-10, PGE2) in tumors. ^*∗*^*p* < 0.05, level of IL-10 and PGE2, CUMS group vs control group.

## Data Availability

The data are all presented in the article.
